# Acetate-buffered crystalloid infusate versus infusion of 0.9% saline and hemodynamic stability in patients undergoing renal transplantation: Prospective, randomized, controlled trial

**DOI:** 10.1007/s00508-017-1180-4

**Published:** 2017-03-02

**Authors:** Carmen Pfortmueller, Georg-Christian Funk, Eva Potura, Christian Reiterer, Florian Luf, Barbara Kabon, Wilfred Druml, Edith Fleischmann, Gregor Lindner

**Affiliations:** 1 Department of Anesthesiology, General Intensive Care Medicine and Pain Management, Medical University of Vienna, Spitalgasse 23, 1090 Vienna, Austria; 2 Department of Respiratory and Critical Care Medicine, Otto Wagner Hospital Vienna and Ludwig-Boltzmann Institute for COPD and Respiratory Epidemiology, Vienna, Austria; 3 Department of Nephrology, Medical University of Vienna, Vienna, Austria; 4 Department of Emergency Medicine, Hirslanden – Klinik Im Park, Zurich, Switzerland

**Keywords:** Balanced, Crystalloid, Hemodynamic, Renal transplantation, Saline

## Abstract

**Background:**

Infusion therapy is one of the most frequently prescribed medications in hospitalized patients. Currently used crystalloid solutes have a variable composition and may therefore influence acid-base status, intracellular and extracellular water content and plasma electrolyte compositions and have a major impact on organ function and outcome. The aim of our study was to investigate whether use of acetate-based balanced crystalloids leads to better hemodynamic stability compared to 0.9% saline.

**Methods:**

We performed a sub-analysis of a prospective, randomized, controlled trial comparing effects of 0.9% saline or an acetate-buffered, balanced crystalloid during the perioperative period in patients with end-stage renal disease undergoing cadaveric renal transplantation. Need for catecholamine therapy and blood pressure were the primary measures.

**Results:**

A total of 150 patients were included in the study of which 76 were randomized to 0.9% saline while 74 received an acetate-buffered balanced crystalloid. Noradrenaline for cardiocirculatory support during surgery was significantly more often administered in the normal saline group, given earlier and with a higher cumulative dose compared to patients receiving an acetate-buffered balanced crystalloid (30% versus 15%, *p* = 0.027; 68 ± 45 µg/kg versus 75 ± 60 µg/kg, *p* = 0.0055 and 0.000492 µg/kg body weight/min, ±0.002311 versus 0.000107 µg/kg/min, ±0.00039, *p* = 0.04, respectively). Mean minimum arterial blood pressure was significantly lower in patients randomized to 0.9% saline than in patients receiving the balanced infusion solution (57.2 [SD 8.7] versus 60.3 [SD 10.2] mm Hg, *p* = 0.024).

**Conclusion:**

The use of an acetate-buffered, balanced infusion solution results in reduced need for use of catecholamines and cumulative catecholamine dose for hemodynamic support and in less occurrence of arterial hypotension in the perioperative period. Further research in the field is strongly encouraged.

## Introduction

Infusion therapy is one of the most frequently prescribed medications in hospitalized patients. The importance of research on fluid therapy has been unrecognized for a long time with normal saline being the most popular infusate [1–4]. During recent years newer balanced infusates containing lactate or acetate became increasingly popular in many European countries whereas in the United States 0.9% saline is still the most widely used infusate in the perioperative and intensive care setting [4]. Currently used crystalloid solutions have a variable composition and may therefore influence acid-base status, intracellular and extracellular water content and plasma electrolyte compositions [5] and have a major impact on organ function and outcome [6]. Despite continuing evaluation no superiority of one particular type of fluid has been achieved so far [7–10]. Nonetheless during recent years it has been shown that the theoretically more physiologically balanced buffered infusion solutions may have a respectable advantage in terms of patient morbidity. A study on healthy human volunteers found a balanced chloride-reduced infusion solution to be associated with better mean renal artery flow velocity, renal cortical tissue perfusion and urine output than infusion of 2 l of 0.9% saline [11]. In a further study including elderly surgery patients, gastric mucosal perfusion was reduced in patients receiving 0.9% saline compared to those receiving chloride-reduced infusion solutions [12]. These data, together with data from rodents with experimental sepsis, which showed significantly lower mean arterial pressure levels when receiving chloride-rich infusion solutions compared to lactated Ringer’s, could suggest an effect of the crystalloid fluid used on a patients’ hemodynamic situation [13].

In the present study we performed a sub-analysis using data from our previously performed prospective randomized controlled trial comparing an acetate-buffered balanced infusion solution compared to 0.9% saline in patients with end-stage renal disease receiving cadaveric renal transplantation [14]. Our aim was to investigate whether use of an acetate-buffered, chloride-reduced balanced infusion solution would result in A.) less need for catecholamine use than use of 0.9% saline and B.) in better hemodynamic stability of patients expressed by the mean arterial blood pressure.

## Material and methods

### Setting

The study was conducted at the Clinic of General Anesthesiology, Intensive Care and Pain Medicine of the Medical University of Vienna.

### Patients, randomization and study fluids

All patients with end-stage renal disease undergoing cadaveric renal transplantation were included in the study. Patients younger than 18 years were excluded from the study as well as patients with a preoperative potassium concentration of more than 5.5 mmol/l. Enrollment started on 1 June 2010 and terminated on 28 February 2013. Prior to enrollment the study was registered at clinicaltrial.org (NCT01075750).

Computer-based randomization was performed at time of transfer to the preoperative care unit of the Department of Anesthesiology. Patients either received normal saline (osmolality 308 mOsm/kg body weight, base excess −24 mmol/l, Na^+^ 154 mmol/L, Cl^−^ 154 mmol/L) or a chloride-reduced, acetate-buffered balanced crystalloid (Elomel Isoton ®, Fresenius Kabi Austria GmbH, Graz; osmolality 302 mOsm/kg, base excess 0 mmol/L, Na^+^ 140 mmol/L, K^+^ 5 mmol/L, Cl^−^ 108 mmol/L, Mg^++^ 1.5 mmol/L, Ca^++^ 2.5 mmol/L, acetate 45 mmol/L). No intravenous fluid was given prior to randomization. The following data were obtained from patients: age, sex, height, actual weight, dry weight, residual daily urine output, number of prior renal transplantations, total of fluid administered intraoperatively, total of fluid at start catecholamines, use of catecholamines, dose of catecholamines per kg body weight per min, time on catecholamines and cumulative vasopressor dose per kg body weight per min.

### Anesthesia

Details on the anesthesia protocol are described elsewhere [14]. After intubation patients were fitted with a central venous line as well as a peripheral venous line.

### Hemodynamic management

Intraoperatively a set infusion rate at 4 ml per kg of ideal body weight per hour (4ml/kg/h) was infused. Hypotension was defined as a mean arterial pressure of less than 60 mm Hg. If hypotension occurred a fluid challenge with 250 ml of infusate was conducted. Additional fluid boluses of 250 ml were administered depending on volume reactivity (increase in mean arterial pressure or/and central venous pressure). If no reaction to volume challenge was seen a vasopressor (either phenylephrine or etilefrine) was administered. A maximum dose of 0.1 mg of phenylephrine and 2 mg of etilefrine was given at one time. If more than ten applications of vasopressor were necessary per hour or likely to exceed ten applications per hour (severe refractory hypotension) noradrenaline infusion was commenced. Additionally, repeated fluid boluses were administered if considered necessary by the anesthesiologist.

### Ethics

The study was approved by the local institutional review board (EK 1048/2009 Oct 2009 and EK 1828/2014 Oct 2014, Chairman Prof. E. Singer), of the Medical University of Vienna, Austria, and registered at a clinical trials registry (NCT01075750). Written informed consent was obtained from every patient included in the study.

### Statistics

The sample size calculation for the original study is described in detail elsewhere [14]. Statistical analysis was performed by SPSS version 17.0 (Chicago, IL). Distribution of interval variables was assessed using normal plots. Interval variables with a normal distribution are presented as means ± standard deviation (SD). Non-normally distributed interval variables and ordinal variables are presented as medians with interquartile ranges (IR). Comparisons of normally distributed interval variables between the saline group and the acetate-buffered balanced crystalloid group were performed using Student’s t‑test. Comparisons of non-normally distributed interval variables and ordinal variables between the saline group and the acetate-buffered balanced crystalloid group were performed using Mann-Whitney U‑test. Comparisons of categorical variables between the saline group and the acetate-buffered balanced crystalloid group were performed by the χ^2^-test.

In order to test whether mean arterial blood pressure differed between the saline group and the acetate-buffered balanced crystalloid group, we used a generalized estimating equation assuming a normal probability distribution and a first order exponential correlation matrix for repeated observations in one patient. In order to test for differences in the incidence of vasopressor administration between the two study groups, we used a log-rank test and a Kaplan-Meier plot for visualization. In order to test whether vasopressor use was anteceded by hypotension, we used a Cox regression with vasopressor use as the dependent variable and mean arterial blood pressure as the time varying predictor variable. For all analyses statistical significance was defined by a two-sided *P* < 0.05.

Figures were drawn using GraphPadPrism 5.01.

## Results

**Fig. 1:**
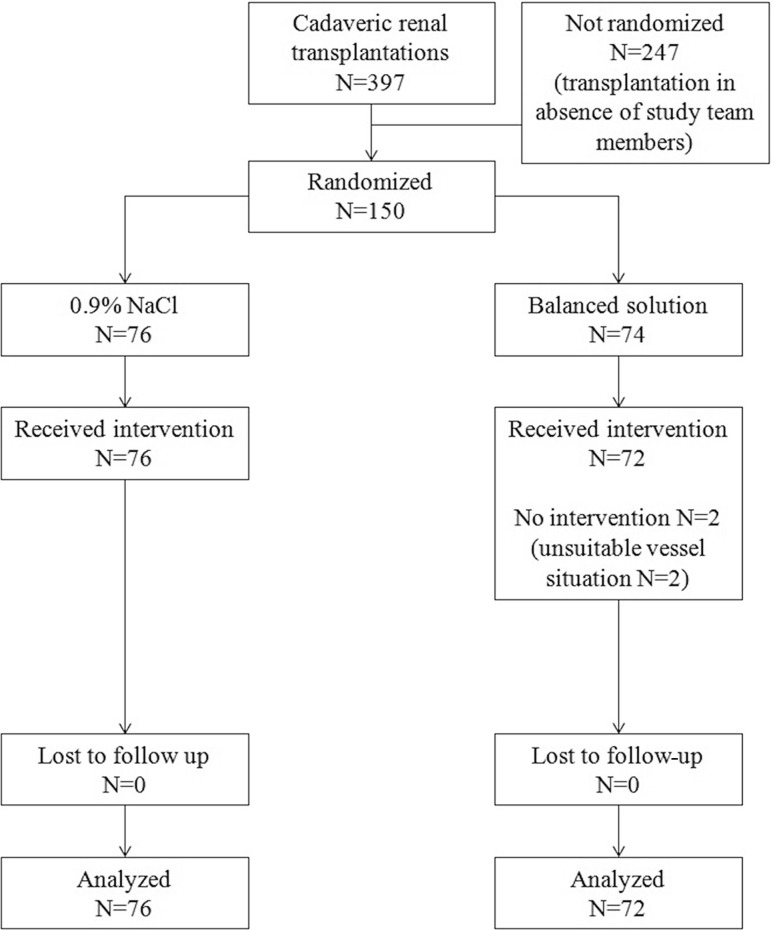
Consolidated Standards of Reporting Trials (CONSORT) flow chart

A total of 150 patients were included in the study, 76 patients were randomized to normal saline and 74 patients to an acetate-buffered balanced crystalloid. No differences between the groups were found for the following parameters: age, sex, height, actual and dry weight, residual daily urine output, number of prior renal transplantations. For an overview on baseline characteristics see Table 1. The CONSORT flow chart is given in Fig. 1.

**Table 1:** Baseline characteristics of patients. Results are mean ± standard deviation or median (1st–3rd quartile)

	Normal saline group			Acetate-buffered balanced crystalloid group			*p-value*
	*Count*	*Percent*		*Count*	*Percent*		
Sex (male/female)	48/28	63/37	–	47/27	64/36	–	0.99
Age (years)	76	100	56 ± 13	74	100	54 ± 13	0.35
Height (cm)	76	100	173 ± 8	74	100	172 ± 10	0.63
Dry weight (kg)	76	100	80 ± 18	74	100	79 ± 16	0.79
Actual weight (kg)	76	100	81 ± 19	74	100	80 ± 16	0.74
Residual urine output (ml/24 h)	76	100	500 (0–1000)	74	100	250 (0–1000)	0.45
Prior kidney transplantation	12	16	–	13	118	–	0.36
Non-heart beating kidney donor	1	1.3	–	2	1.4	–	0.51
Postoperative necessity for hemodialysis	19	25	–	19	25.7	–	0.49

**Fig. 2:**
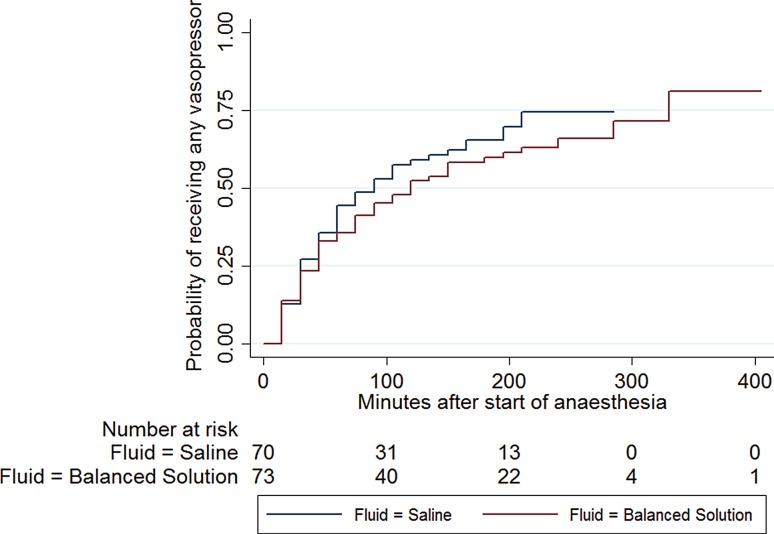
Kaplan Meyer analysis: time to catecholamines

In the 0.9% saline group the mean volume of fluid received during surgery was 1691 ± 664 ml compared to 1798 ± 679 ml in the balanced acetate-based infusate group (*p* = 0.34). Noradrenaline for cardiocirculatory support during surgery was administered significantly more often in the normal saline group compared to patients receiving an acetate-buffered balanced crystalloid (30% versus 15%, *p* = 0.027). Patients receiving normal saline needed noradrenaline significantly earlier and the cumulative noradrenaline dose was significantly higher than patients receiving an acetate-based crystalloid solute (68 min ±45 versus 75 min ±60, *p* = 0.0055 and 0.000492 µg/kg body weight/min, ±0.002311 versus 0.000107 µg/kg/min, ±0.00039, *p* = 0.04, respectively). No difference in necessity for vasopressor or cumulative dose of vasopressors was seen between the groups (*p* = 0.47 and *p* = 0.08, respectively) (see Table 2; Fig. 2).

**Table 2:** Catecholamines and vasopressors needed in the groups studied (*n*, percentages, mean and standard deviations)

Normal saline group			Acetate-buffered balanced crystalloid group		*P-value*
*Duration of anesthesia*	184	±73	166	±77	0.15
Total intraoperative fluid (ml)	1691	±664	1798	±679	0.34
*Vasopressor*	56	73.7%	50	67.6%	0.47
Cumulative vasopressor dose (µg/kg/min)	0.00011	±0.00017	0.000083	±0.000015	0.08
*Catecholamines*	23	30%	12	15%	0.027
Fluid until vasopressor (ml)	407	±287	735	±482	0.007
Time to catecholamines (min)	68	±45	75	±60	0.0055
Cumulative catecholamine dose (µg/kg/min)	0.000492	±0.002311	0.000107	±0.00039	0.04
Time on catecholamines (min)	141	74	175	75	0.6

**Fig. 3:**
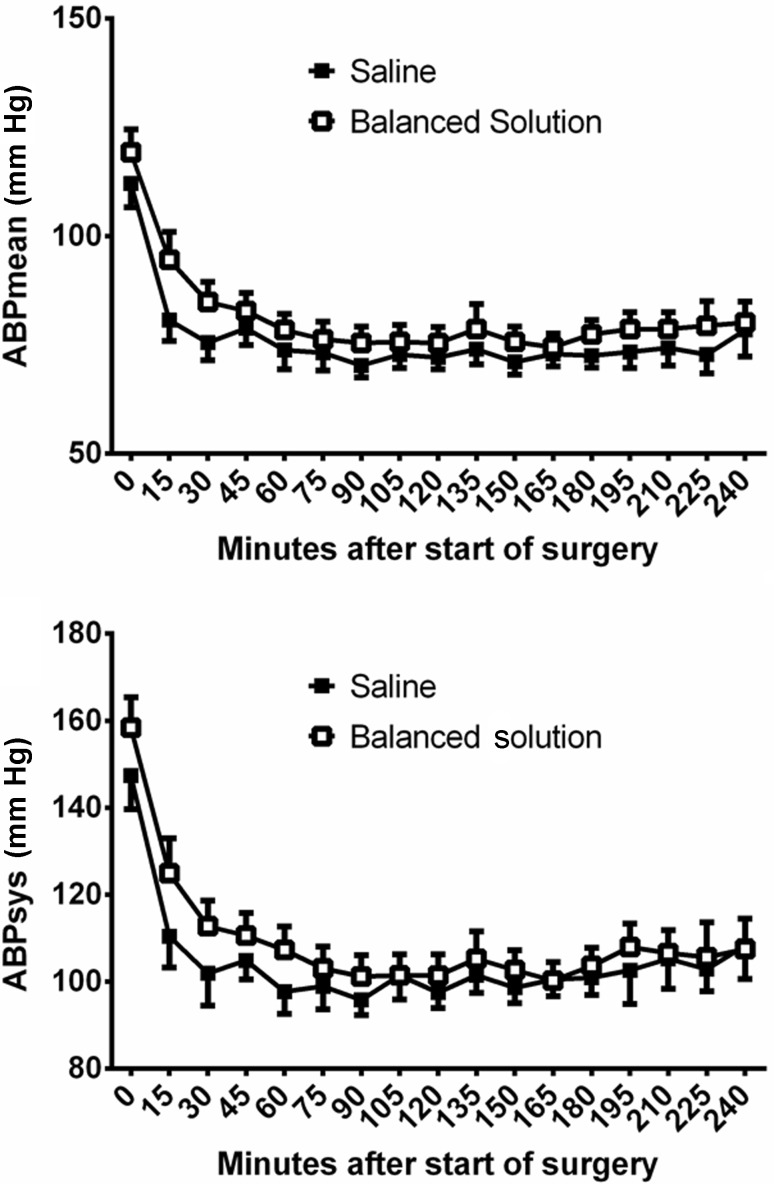
Mean (*upper panel*) and systolic (*lower panel*) blood pressure levels in patients receiving either 0.9% saline or an acetate-buffered balanced infusion solution

**Fig. 4:**
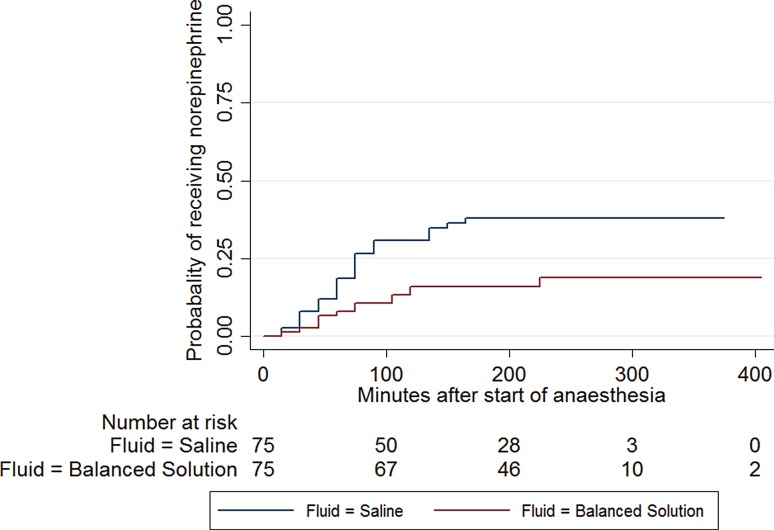
Kaplan Meier analysis: probability to need norepinephrine

Mean minimum arterial blood pressure was significantly lower in patients randomized to 0.9% saline than in patients receiving the balanced infusion solution (57.2 [SD 8.7] versus 60.3 [SD 10.2] mm Hg; *p* = 0.024). Consequently, mean minimum systolic (77.7 [SD 17.1] versus 80.1 [SD 16.6] mm Hg; *p* = 0.0398) and mean minimum diastolic blood pressure (43.6 [SD 7.2] versus 45.7 [SD 8.6] mm Hg; *p* = 0.0485) were significantly lower in patients randomized to 0.9% saline. Patients randomized to 0.9% saline over time had a mean arterial blood pressure, which was 4 mm Hg lower than in patients receiving an acetate-buffered balanced solution during whole surgery (95% CI: 1–7 mm Hg; *p* = 0.009). Fig. 3 gives mean and systolic blood pressure levels during surgery for patients receiving either 0.9% saline or an acetate-buffered balanced infusion solution. In the Cox regression analysis it could be shown that patients receiving catecholamines had significantly lower blood pressure levels before administration of catecholamines than patients without catecholamines (see Fig. 4). There was no difference in heart rate (normal 73 beats per minute ±12.6 versus acetate-buffered solution 74 beats per minute ±12.2, *p* = 0.66) and central venous pressure (normal saline 9 ± 10 mmH_2_O, acetate-buffered solution 8 ± 5 mmH_2_O, *p* = 0.34) between the groups.

Peak chloride levels were significantly higher in patients randomized to the normal saline group (109 mmol/L [107 to 111] versus 107 mmol/L [105 to 109]), *p* = 0.001 as was fluctuation in serum chloride levels during surgery (4 mmol/L [3 to 6] versus 3 mmol/L [2 to 5]), *p* = 0.03. Serum sodium levels were not significantly different between the groups (4 mmol/L [3 to 5] versus 3 mmol/L [2 to 5]), *p* = 0.92. Acid base disturbances are described in more detail elsewhere [14].

## Discussion

These are the first data from a prospective, randomized, controlled study comparing the effects of 0.9% saline and an acetate-buffered balanced crystalloid solution on hemodynamics in the perioperative period. We showed that perioperative use of a balanced crystalloid as a maintenance fluid is associated with better hemodynamic stability of patients with end-stage renal disease receiving cadaveric renal transplantation. These findings are expressed by a significantly lower need for use of catecholamines for hemodynamic support and higher systolic and mean arterial blood pressure levels.

The strength of the current study is explained by the study collective: all patients had end-stage renal disease and received cadaveric renal transplantation. This collective of patients is especially vulnerable to effects due to infusion solutions since they lack the capacity of the kidneys to rapidly compensate for electrolyte and acid-base derangements to a variable degree. Theoretically, this makes even slight effects due to the infusion solution obvious, which would not be seen in patients with normal kidney function or only after administration of far larger volumes of fluid.

We found a significantly lower need for catecholamines, a longer time to catecholamines and a lower cumulative catecholamine dose in the acetate-buffered group. The significantly better hemodynamic stability in patients receiving a balanced acetate-based infusate compared to normal saline might be related to A) acetate itself and B) hyperchloremia induced by normal saline.

So far no study has compared the hemodynamic effects of normal saline to an acetate-buffered balanced crystalloid; however, there is some older evidence that the use of sodium acetate may have an influence on the cardiovascular system. In 1978 Liang and Lowenstein infused acetate and pyruvate in to anesthetized dogs to measure their impact on circulation [15]. They found that increased acetate levels were associated with a significant increase in cardiac output [15]. Even though myocardial oxygen consumption increased during acetate infusion, the decrease in myocardial oxygen uptake and the increase in coronary sinus blood oxygen saturation suggest that an active coronary vasodilation which was not a result of the increased cardiac work takes place [15]. Acetate infusion also increased blood flow to the gastrointestinal tract, kidneys, intercostal muscles and diaphragm [15]. In another study by Conahan et al. on resuscitation fluid composition and myocardial performance during burn shock in guinea pigs, the authors were able to show that treatment with Ringer’s acetate significantly improved cardiac output and contractility when compared to normal saline and Ringer’s lactate [16]. In the same study Ringer’s acetate was found to have better blood pressure stabilizing effects compared to Ringer’s lactate and normal saline [16]. Concerning blood pressure stabilization three studies on sodium acetate found that such an infusion may have a positive effect on cardiac output but it lowers peripheral vascular resistance [17–19], a finding that we cannot confirm; however, it has to be born in mind that data on acetate and acetate-buffered crystalloid solutes and hemodynamics remain scarce and are mostly experimental. There is a lack of controlled randomized trials in humans that compare currently available crystalloid solutes with respect to hemodynamics and further research certainly should be encouraged.

In 2004 the study group around John Kellum showed in a model of experimental sepsis that infusion of chloride-rich crystalloids led to a decrease in arterial blood pressure in rodents compared to infusion of lactated Ringers [13]. In another cross-over study on 12 healthy volunteers Chowdhury et al. found that a colloid, embedded in a balanced crystalloid solution, led to increased renal cortical tissue perfusion while the same concentration of colloid, embedded in 0.9% saline, did not result in improved renal perfusion [20]. A similar trial of this group in which 2 l of 0.9% saline or a chloride-reduced, balanced crystalloid was administered to healthy volunteers showed that administration of 0.9% saline even resulted in a decline in renal blood flow velocity and renal cortical tissue perfusion [11]. Given this evidence, together with other recent studies on this subject [2, 21, 22], it is plausible that hyperchloremia induced by infusion of chloride-rich solutions, is the direct trigger for unfavorable hemodynamic effects, as seen in our current study. Only recently, large scale studies and meta-analysis showed that use of chloride-rich infusion solutions might be associated with adverse outcome [2, 23, 24]; however, a Cochrane review from 2012 still came to the conclusion that although balanced crystalloids are associated with less occurrence of hyperchloremia and concurrent metabolic acidosis, use of conventional solutions (i. e. 0.9% saline) can be considered safe in the perioperative period [25]. Additionally, a recently published randomized controlled trial comparing 0.9% saline to an acetate-buffered crystalloid solute found no differences in terms of acute kidney failure and renal replacement therapy [26]. Given the current data together with previously published studies in the field, the need for prospective randomized controlled trials is obvious. While studies comparing the currently available crystalloid solutes with mortality as the primary endpoint will be hard to perform due to the large patient numbers needed and probable issues with the financing of such a study, it seems applicable to perform studies focusing on patient hemodynamics or renal function.

## Limitations

Our study has several limitations. The major drawback of this study is the lack of invasive hemodynamic monitoring such as esophagus Doppler, pulmonary artery catheter or thermodilution techniques. Additionally, blood pressure was monitored non-invasively, which was performed in order to preserve blood vessels in patients potentially in need of dialysis shunts in the future. Additionally, it has to be borne in mind that the original study was designed to investigate incidence rates of perioperative hyperkalemia, other electrolyte and acid-base disorders and not hemodynamics. Therefore, hemodynamic management followed hospital standard operating protocols (SOP but not a hemodynamic protocol specially designed for the present study. Additionally, the study was not blinded, so investigator bias is possible but not very likely as hemodynamics were not an outcome in the first study and hospital SOP’s were followed. Nonetheless the results of the present study should be interpreted with some caution. We see our results as a first indiction that the choice of crystalloid may impact hemodynamic stability, but certainly further research needs to be done before an overall conclusion can be reached.

## Conclusion

In conclusion, the results of the prospective, randomized, controlled trial suggest that use of an acetate-buffered, balanced infusion solution results in less occurrence of arterial hypotension and reduced need for use of catecholamines and cumulative catecholamine dose for hemodynamic support in the perioperative period. The more favorable hemodynamic outcome of patients receiving an acetate-buffered crystalloid solute may be attributed to increased cardiac output related to acetate as well as lower susceptibility for hyperchloremia and concurrent metabolic acidosis when compared to normal saline. Future randomized, controlled trials are certainly desirable to clarify the effects of current crystalloid infusates on hemodynamics in the perioperative field and the critically ill.
